# Exercise characteristics and incidence of abnormal electrocardiogram response in long‐distance runners with exercise‐induced hypertension

**DOI:** 10.1111/jch.14359

**Published:** 2021-08-29

**Authors:** Young‐Joo Kim, So‐Eun Lee, Kyoung‐Min Park

**Affiliations:** ^1^ Department of Exercise Rehabilitation Welfare Soojung Campus Sungshin Women's University Seoul Republic of Korea; ^2^ College of Wesley Creative Convergence Hyupsung University Gyeonggi‐do Republic of Korea; ^3^ Division of Cardiology Department of Medicine Samsung Medical Center Sungkyunkwan University School of Medicine Seoul Republic of Korea

**Keywords:** arrhythmia, electrocardiogram, exercise, hypertension, runner

## Abstract

While long‐distance running has important health benefits, chronic elevation of blood pressure during exercise might induce cardiac events and sudden death. This study aimed to investigate characteristics of exercise and incidence of abnormal exercise electrocardiography findings in long‐distance runners with exercise‐induced hypertension. Long‐distance runners (*n* = 606) underwent a questionnaire survey, history taking, and exercise stress testing, and they were classified into the non‐exercise‐induced (*n* = 268) and exercise‐induced (*n* = 338) hypertension groups. Exercise‐induced hypertension was defined as systolic blood pressure ≥210 mm Hg during maximal exercise. Abnormal electrocardiogram response (AER) were defined as abnormal electrocardiography findings, such as arrhythmias or ST‐segment changes, during exercise stress testing. There were no differences in general and exercise‐related characteristics between the non‐exercise‐induced and exercise‐induced hypertension groups. The AER group (AERg, *n* = 37) had a significantly longer training history and total exercise time than the non‐AER group (non‐AERg, *n* = 569) (*p *< .05). Atrial arrhythmias and ST‐segment depression were more prevalent in the exercise‐induced hypertension group than in the non‐exercise‐induced hypertension group (atrial arrhythmias: 5% [17/338] vs. 1.9% [5/268]; ST‐segment depression: 2.7% [9/338] vs. .4% [1/268]; *p *< .05). The incidence of AER was significantly higher in the exercise‐induced hypertension group (*n* = 30, 8.8%) than in the non‐exercise‐induced hypertension group (*n* = 7, 2.6%) (*p *< .05). This study showed that long‐distance runners with AER had a longer training history and total exercise time than those without AER, and the exercise‐induced hypertension group had a higher rate of AER.

## 1 INTRODUCTION

Regular exercise and physical activity decrease the risk of cardiovascular events (CE), chronic heart disease, and mortality.^1^, ^2^ Therefore, long‐distance runners, such as half‐marathon or full‐marathon runners, are at a low risk of heart attack and sudden death,^3^ and walking and moderate‐intensity running lower the incidence of arrhythmia.^4^ However, endurance athletes may be vulnerable to fatal arrhythmias, such as atrial fibrillation (AF), atrial flutter, and ventricular tachycardia.^5^, ^6^, ^7^ Indeed, the incidence of AF in endurance athletes is reported to be five‐fold higher than that in the general population.^8^, ^9^ For example, a study found that among 50 middle‐aged male long‐distance runners, 50% had coronary artery stenosis, and 6% had a vessel with > 50% diameter stenosis.^10^

Recently, a variety of CE have been reported in long‐distance runners with exercise‐induced hypertension (EIH). Runners with EIH have weakened ventricular diastolic functions,^11^ with marked elevation of myocardial infarction and heart failure markers, such as cardiac troponin I and N‐terminal pro b‐type natriuretic peptide, following long‐distance running.^12^, ^13^ In particular, runners with EIH show a high incidence of coronary artery plaque,^14^ suggesting that EIH may be a risk factor for sudden death in long‐distance runners.

EIH is defined as systolic blood pressure (SBP) ≥210 mm Hg for men and ≥190 mm Hg for women during exercise.^15^, ^16^ Excessive elevation of blood pressure during exercise is a risk factor for cerebral and cardiovascular diseases,^17^, ^18^ with a high likelihood of progressing to hypertension at rest.^19^ Runners exhibiting excessive elevation of blood pressure during exercise would have a high rate‐pressure product (RPP) due to the high volume pressure on the atria and ventricles. Because excessive elevation of blood pressure during every exercise session chronically strains the cardiac muscles, the incidence of abnormal electrocardiogram response (AER) such as arrhythmia or myocardial ischemia is anticipated to rise. Therefore, more exercise is not always good. The present study investigated the incidence of AER by examining abnormal exercise electrocardiography (ECG) findings in long‐distance runners with EIH to make an indirect prediction of CE.

## 2 METHODS

### 2.1 Participants and study protocol

**FIGURE 1:**
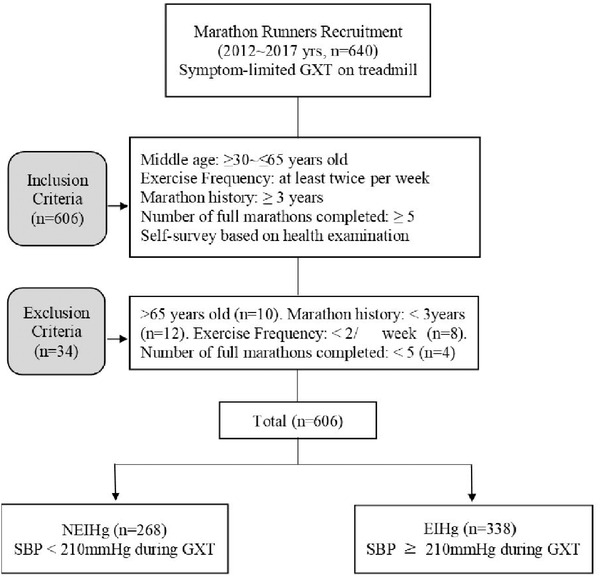
Flow chart of the study procedure. GXT, graded exercise test; NEIHg, non‐exercise‐induced hypertension group; EIHg, exercise‐induced hypertension group; SBP, systolic blood pressure

The study protocol is illustrated in Figure 1. A total of 640 long‐distance runners were enrolled in this study. A questionnaire survey and individual history taking were performed with reference to their clinical and exercise characteristics and medication history, as well as annual health examination results. All enrolled runners underwent a symptom‐limited graded exercise test (GXT). The inclusion criteria were as follows: amateur marathoners aged between 30 and 64 years, marathon history of 3 years or longer, exercise frequency of ≥ twice/week, and at least five full marathons completed. Of the 640 participants, 34 did not meet these criteria and were excluded. Exercise‐induced hypertension group (EIHg) is defined as participants whose SBP increases ≥ 210 mm Hg during the maximal exercise test, regardless of whether they have hypertension during resting or not, and non‐exercise‐induced hypertension group (NEIHg) is also defined as participants whose SBP increases < 210 mm Hg during the test, regardless of presence of hypertension during resting.

The RPP is a measure of the stress placed on the cardiac muscle based on the number of times needed to beat per minute and the arterial blood pressure, that is, pumping against. The RPP was calculated by multiplying SBP by the heart rate. Informed consent was obtained from all the patients before enrollment in the study. This study complied with the Declaration of Helsinki, and the research protocol was approved by the Sungshin Women's University Institutional Ethics Board (No. SSWUIRB‐2020‐048).

### 2.2 GXT

Heart rate and blood pressure were measured at rest, during exercise, and at 5 min after recovery. ECG was recorded at rest, once at 1 min before the end of each stage, every 1 min during recovery, and continuously during cardiac events, such as myocardial ischemia. The Bruce protocol was as follows: VO_2max_ was measured using a treadmill (T170DE, HP cosmos, Traunstein, Germany) and a respiratory gas analyzer (Quark CPET, Cosmed, Lazio, Italy), and arrhythmia and myocardial ischemia were monitored using an ECG (CH2000, Cambridge Heart, MA, USA). Blood pressure was measured using an automated blood pressure monitor system (Tango+, Suntech, NC, USA), and a high‐technology microphone was attached to the brachial artery to directly listen to the sounds using a headphone. The Borg scale (6–20) was used to measure the rated perceived exertion (RPE), which represents perceived exertion with increasing levels of exertion during GXT.^20^

### 2.3 Abnormal ECG response

Participants were classified into the AER group (AERg) and non‐AER group (non‐AERg) according to the presence of abnormal exercise ECG, such as arrhythmias or ST‐segment changes. Atrial arrhythmias were frequent atrial premature contractions (APCs), AF, and atrial flutter (AFL). AF/AFL included both AF/AFL observed during the GXT and AF/AFL diagnosed based on a historical review, and frequent APC was defined as two or more APCs per 10 s (sec).^21^ Non‐sustained ventricular tachycardia (NSVT) was defined as three or more premature ventricular contractions within 30 s.^20^ Those parameters mentioned above and ST‐segment depression (STD) (upslope type ≥ 2 mm, horizontal type ≥ 1.4 mm, and downslope type ≥ 1 mm) were determined in accordance with the American College of Cardiology/American Heart Association guidelines.^20^

### 2.4 Statistics

Between‐group analysis was performed using kurtosis and skewness of the data and the Kolmogorov–Smirnov test. Based on the results, an independent t‐test was used as a parametric method, and the Mann–Whitney U test was used as a nonparametric method. A 2 × 2 crosstabulation analysis was performed to analyze the percentages between the groups. The significance level was set at 0.05, and statistical analyses were performed using SPSS version 21.

## 3 RESULTS

### 3.1 Baseline characteristics

There were no differences in general characteristics between the NEIHg and EIHg. There were no statistically significant differences in prevalence of hypertension between groups (NEIHg, 7.8% vs. EIHg, 11.2%). Regarding hemodynamic characteristics, heart rate at rest, blood pressure, and HRmax were similar between the two groups, while maximum SBP and DBP were significantly higher in the EIHg than in the NEIHg (SBP: 234.8 ± 17.0 vs. 191.0 ± 13.3; DBP: 76.4 ± 14.5 mm Hg vs. 72.3 ± 11.1 mm Hg) (*p *< .05; Table 1). Three SBP measurements taken every minute during the recovery after the maximal exercise decreased over time, with consistently significantly higher values in the EIHg than in the NEIHg (SBP recovery at 1 min: 219.3 ± 24.5 vs. 177.4 ± 19.8 mm Hg; SBP recovery at 2 min: 206.9 ± 25.3 vs. 178.4 ± 20.1 mm Hg; and SBP recovery at 3 min: 198.2 ± 25.0 vs. 170.1 ± 20.0 mm Hg; *p *< .05). Regarding physical performance, there were no significant differences in maximum oxygen uptake, total exercise time, or maximal metabolic equivalents between the two groups.

**TABLE 1:** Demographics, hemodynamic characteristics, and cardiorespiratory fitness of study participants

	NEIHg (*N* = 268, 44%)	EIHg (*N* = 338, 56%)	Total (*N* = 606)
*General characteristics*			
Age (years)	48.4±7.1	49.9±7.2	49.2 ±7.2
Height (cm)	169.5±8.0	170.2±5.3	169.9 ±6.6
Weight (kg)	67.5±6.9	67.9±7.2	67.8 ±7.1
BMI (kg/m^2^)	23.3±1.9	23.4±2.0	23.4 ±2.0
Smoking status (%)	25 (9.3)	31 (9.2)	56 (9.2)
Alcohol (%)	179 (66.8)	237 (70.1)	416 (68.6)
Hypertension (%)	21 (7.8)	38 (11.2)	59 (9.7)
DM (%)	4 (1.5)	11 (3.3)	15 (2.5)
Hyperlipidemia (%)	5 (1.9)	2 (0.6)	7 (1.2)
*Hemodynamic characteristics*			
HR_rest_ (BPM)	65.0 ±10.2	64.1 ±9.5	64.5 ±9.8
HR_max_ (BPM)	174.2 ±11.8	172.6 ±13.5	173.3 ±12.8
SBP_rest_ (mm Hg)	128.4 ±13.3	136.3 ±14.2	132.8 ±14.4
DBP_rest_ (mm Hg)	84.5 ±9.4	83.8 ±10.0	84.1 ±9.7
SBP_max_ (mm Hg)	191.0 ±13.3	234.8 ±17.0^*^	215.4 ±26.7
DBP_max_ (mm Hg)	72.3 ±11.1	76.4 ±14.5^*^	74.6 ±13.3
RSBP1 (mm Hg)	177.4 ±19.8	219.3 ±24.5^*^	200.7 ±30.6
RSBP2 (mm Hg)	178.4 ±20.1	206.9 ±25.3^*^	194.3 ±27.1
RSBP3 (mm Hg)	170.1 ±20.0	198.2 ±25.0^*^	185.7 ±26.8
*Physical performance*			
VO_2max_ (ml/kg/min)	50.1 ±6.9	49.7 ±7.1	49.9 ±7.0
Total Ex. Time (s)	790.1 ±101.4	795.2 ±96.3	793.0 ±98.5
METs_max_	14.3 ±1.9	14.2 ±2.0	14.2 ±2.0

Data are presented as mean ± SD.

*Abbreviations*: NEIHg, non exercise‐induced hypertension; EIHg, exercise‐induced hypertension group; BMI, body mass index; DM, diabetes mellitus; HR, Heart Rate; BPM, beat per minute; SBP, systolic blood pressure; DBP, diastolic blood pressure; RSBP, recovery systolic blood pressure; Total Ex. Time, Total exercise time; METs, maximal metabolic equivalents.

*Significant difference between the NEIHg and EIHg at *p* < .05.

### 3.2 Exercise characteristics

Exercise characteristics of included marathoners are shown in Table 2. Approximately 56% (338 of 606) of the participants were allocated in the EIHg. There were no significant differences in training history, marathon time, marathon completion, exercise frequency, or exercise intensity between the NEIHg (*n* = 268) and EIHg (*n* = 338).

**TABLE 2:** Exercise characteristics of included marathoners

Exercise data	NEIHg (*N* = 268, 44%)	EIHg (*N* = 338, 56%)	Total (*N* = 606)
Training history (month)	99.2 ±64.4	99.7 ±56.9	99.5 ±60.3
Marathon time (min)	219.8 ±34.1	216.1 ±32.8	217.8 ±33.4
Exercise time (min/day)	100.2 ±39.9	104.4 ±41.0	102.5 ±40.5
Total exercise time (min)	2627.2 ±2716	2819.0 ±2745.3	2734.1 ±2732.0
Marathon completed (number)	46.7 ±46.2	46.5 ±52.1	46.6 ±49.5
Exercise frequency (times per week)	3.6 ±1.2	3.8 ±1.3	3.7 ±1.3
Exercise intensity (Borg's RPE scale)	12.9 ±1.8	13.3 ±1.6	13.1 ±1.7

Data are presented as mean ± SD.

*Abbreviations*: NEIHg, Non exercise‐induced hypertension group; EIHg, Exercise‐induced hypertension group; RPE, rated perceived exertion. .

Participants with AER accounted for 5.9% of all the participants. The AERg had a significantly longer training history (116.7 ± 75.8 months vs. 98.4 ± 59.0 months; *p *< .05) and a significantly longer total exercise time (3594.9 ± 3965.5 min vs. 2678.2 ± 2627.2 min; *p *< .05) than the Non‐AERg. There were no significant differences in marathon time, marathon completion, exercise frequency, exercise intensity, or VO_2max_ between the AERg and Non‐AERg (Table 3).

**TABLE 3:** Exercise characteristics and VO_2max_ in the abnormal ECG response group

Exercise data	Non‐AERg (*N* = 569)	AERg (*N* = 37)
Training history (month)	98.4 ±59.0	116.8 ±75.7^*^
Marathon time (min)	218.0 ±33.6	214.1 ±30.0
Exercise time (min/day)	102.7 ±40.8	100.0 ±36.0
Total exercise time (min)	2678.2 ±2627.2	3594.9 ±3965.5^*^
Marathon completed (number)	46.2 ±48.6	53.3 ±63.3
Exercise frequency (times per week)	3.7 ±1.2	4.2 ±1.3
Exercise intensity (Borg's RPE scale)	13.1 ±1.7	13.6 ±1.6
VO_2max_, ml/kg/min	49.9 ±7.0	49.5 ±6.5

Data are presented as mean ± SD.

*Abbreviations*: ECG, electrocardiogram; Non‐AERg, non‐abnormal ECG response group; AERg, abnormal ECG response group; RPE, rated perceived exertion.

*Significant difference between the Non‐AERg and AERg at *p* < .05.

**FIGURE 2:**
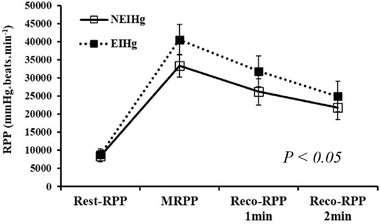
RPP at rest, maximum exercise, and recovery in the GXT. GXT, graded exercise test; NEIHg, non‐exercise‐induced hypertension group; EIHg, exercise‐induced hypertension group; RPP, rate pressure product; MRPP, maximum rate pressure product; Reco, recovery; *, significant difference between NEIHg and EIHg at *p *< .05

RPP at rest did not significantly differ between the two groups (NEIHg vs. EIHg: 8365.7 ± 1617.7 vs. 8739.0 ± 1602.3 mm Hg_*_beats_*_min^−1^), but RPP at maximal exercise, 1 min into recovery, and 2 min into recovery were significantly higher in the EIHg than in the NEIHg (NEIHg vs. EIHg: 33280.1 ± 3164.5 vs. 40526.4 ± 4248.7 mm Hg_*_beats_*_min^−1^, 26176.5 ± 3654.5 vs. 31772.7 ± 4558.3 mm Hg_*_beats_*_min^−1^, and 21811.9 ± 3303.1 vs. 24881.8 ± 4218.6 mm Hg_*_beats_*_min^−1^, respectively) (Figure 2).

### 3.3 Abnormal ECG response

**FIGURE 3:**
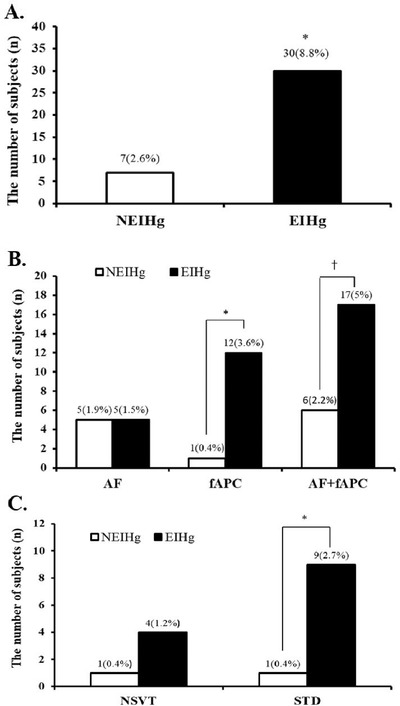
(A) Total cardiac events response, (B) Atrial arrhythmias event, (C) NSVT and ST‐segment depression events. NEIHg, non‐exercise‐induced hypertension group; EIHg, exercise‐induced hypertension group; AF, atrial fibrillation; fAPC, frequent atrial premature contraction; NSVT, non‐sustained ventricular tachycardia; STD, ST segment depression; *, significant difference between the NEIHg and EIHg at *p* < .05

The incidence of AF did not significantly differ between the NEIHg (*n* = 5, 1.9%) and EIHg (*n* = 5, 1.5%). However, the incidence of frequent APC was significantly higher in the EIHg group (*n* = 12, 3.6%) than in the NEIHg group (*n* = 1, 0.4%) (*p *< .05). Overall, the incidence of atrial arrhythmia was significantly higher in the EIHg group (*n* = 17, 5%) than in the NEIHg group (*n* = 5, 1.9%) (*p *< .05) (Figure 3).

While the incidence of NSVT did not significantly differ between the NEIHg (*n* = 1, 0.4%) and EIHg (*n* = 4, 1.2%), the incidence of STD was significantly higher in the EIHg (*n* = 9, 2.7%) than in the NEIHg (*n* = 1, 0.4%) (*p *< .05) (Figure S1). In addition, the rate of AER was significantly higher in the EIHg group (*n* = 30, 8.8%) than in the NEIHg group (*n* = 7, 2.6%) (*p *< .05) (Figure S2).

## 4 DISCUSSION

This study investigated the incidence of abnormal exercise ECG response and the factors related to exercise history in long‐distance runners with EIH to determine whether EIH is a risk factor for AER. Of the 606 long‐distance runners included in this study, 44% were in the NEIHg with normal blood pressure response during the maximal exercise, while 56% were in the EIHg with EIH during the maximal exercise. Notably, more than half of the long‐distance runners had EIH, and their SBP remained high even during the recovery phase after the maximal exercise. While the two groups did not differ in exercise characteristics, the training history was approximately 18 months longer in the group with AER, such as AF/AFL, frequent APC, NSVT, and STD, than in the group with non‐CE. Hence, EIH might be due to cardiac overload during and after exercise.

AF has been observed in athletes with a training history of ≥ 10 years at ≥ 3 h training per week^22^ and those with more than 1500 h of training.^8^ The etiological mechanism of AF in long‐distance runners involves increased vagal tone caused by excessive chronic exercise that stretches the atria; atrial enlargement is accompanied by inflammation and atrial scar formation during healing progresses to fibrosis, thereby causing AF.^6^ Based on this hypothesis, high atrial volume pressure during exercise in runners with EIH would accelerate the onset of AF.

Although EIH is an independent risk factor for cerebral and cardiovascular diseases,^17,18^ Joint National Committee‐8 only presents an algorithm for blood pressure at rest and does not take into account EIH.^23^ Recently, Kim and coworkers^14^ demonstrated that long‐distance runners with EIH have a higher rate of coronary artery plaque formation on multi‐detector computed tomography (MDCT) than runners with normal SBP during exercise. The present study also observed that ST‐segment depression positivity, which indicates myocardial ischemia, was significantly higher in the EIHg during the GXT, suggesting that EIH may be another risk factor in long‐distance runners. The mechanism which runners with exercise‐induced hypertension have more prevalence of ST‐segment depression is that increased oxidative stress by chronical(prolonged) excessive blood pressure elevation during running induces endothelial dysfunction in the coronary arteries, which could increase plaque formation in coronary arteries.^24^ EIH is induced by elevated afterload due to impaired peripheral vascular contractility, which in turn leads to excessive elevation of blood pressure.^25^, ^26^ Long‐distance runners are exposed to such chronic excessive elevation of blood pressure for longer periods than the general population, even if their blood pressure is normal at rest. Indeed, the amount of exercise in marathoners is 5–10 times higher than that of exercise recommended in the guidelines for the cardiovascular disease prevention, and even marathoners without EIH have a higher coronary artery calcium score than the general population.^27^ If runners with EIH have potential myocardial ischemia, as shown in our study, the excessive elevation of RPP would be further promoted during exercise. Considering that a common cause of sudden death is congenital hypertrophic cardiomyopathy in athletes aged 35 years or younger and acquired coronary artery disease in those aged 35 years or older,^28^ EIH might further facilitate the onset of coronary artery diseases in middle‐aged long‐distance runners. Consistently, the overall incidence of abnormal ECG response was also significantly higher in the EIHg than in the NEIHg. Even though the AER is an indirect prediction of various CEs, this finding suggests that EIH may be a new risk factor for sudden death in long‐distance runners. Although excessive exercise poses a risk of sudden death, physical activity should still be encouraged because the health benefits outweigh the risk,^29^ and the desire for such exercise should not be suppressed because the risk of mortality does not increase even with excessive exercise 10 times greater than the amount of recommended exercise.^30^ However, periodic non‐invasive MDCT or GXT should be performed for middle‐aged runners with EIH, and potential therapeutic targets should be further identified. Notably, angiotensin II receptor blockers have recently been proposed to be effective for EIH because EIH is associated with the marked activation of angiotensin II in the renin‐angiotensin‐aldosterone system. However, further clinical studies are needed to determine their potential clinical applications.^31^, ^32^ Taken together, this study suggests that EIH may be a risk factor for AER, such as arrhythmia and coronary artery disease, during exercise in long‐distance runners. The findings necessitate aggressive diagnostic testing for early detection and prevention of cerebral and cardiovascular diseases.

This study has several limitations. First, we could not assess family history, which might have affected EIH. Second, we could not investigate whether the participants engaged in additional exercise other than long‐distance running, which might have affected their exercise characteristics. Third, the self‐reported exercise time per session, exercise frequency, and exercise intensity were only approximate data obtained based on a questionnaire survey. Fourth, the participants might have had undiagnosed metabolic disorders, such as hypertension, diabetes mellitus, and hyperlipidemia. Fifth, we only analyzed questionnaire survey results, history taking results, and single abnormal ECG responses that were observed during GXT, but we could not conduct laboratory analysis, 24‐h Holter ambulatory monitoring, echocardiography, and MDCT; therefore, adverse events might have been underestimated. However, the findings of this study are still relevant because we found a significantly higher incidence of cardiac events in the EIHg despite the above limitations.

## 5 CONCLUSIONS

This study found that long‐distance runners with AER had a longer training history and total training time than their counterparts without AER, and the incidence of AER, such as atrial arrhythmias and STD, was higher in the EIHg than in the NEIHg.

## CONFLICT OF INTEREST

The authors have indicated that they have no conflicts of interest regarding the content of this review paper.

## AUTHOR CONTRIBUTION

Conceptualization, Kyoung‐Min Park; data curation, So Eun Lee and Young‐Joo Kim; formal analysis, Kyoung‐Min Park and Young‐Joo Kim; supervision, Kyoung‐Min Park; writing—original draft, Young‐Joo Kim and So Eun Lee; writing—review and editing, Kyoung‐Min Park All authors have read and agreed to the published version of the manuscript.
